# Real-world utilization patterns and survival in men with metastatic prostate cancer treated with Radium-223 in the United States

**DOI:** 10.1038/s41391-025-00969-6

**Published:** 2025-04-04

**Authors:** Amit D. Raval, Yiqiao Zhang, Matthew Korn, Niculae Constantinovici, Rana R. McKay

**Affiliations:** 1 Bayer HealthCare Pharmaceuticals, Whippany, NJ USA; 2 Bayer Consumer Care, Basel, Switzerland; 3 Division of Hematology-Oncology, Department of Medicine, University of California, San Diego, La Jolla, CA USA

**Keywords:** Cancer therapy, Prostate cancer

## Abstract

**Background:**

The treatment landscape for metastatic castration-resistant prostate cancer (mCRPC) has evolved since radium-223 (Ra-223) was approved in the United States (2013). We examined treatment patterns and real-world overall survival (rwOS) of men with mCRPC treated with Ra-223 in the modern treatment era.

**Methods:**

A retrospective cohort of men treated with Ra-223 was derived using private insurance data from the Komodo Health dataset from January 1, 2017 to June 30, 2022. Cox-regression analyses examined associations between Ra-223 use and rwOS with adjustment for covariates.

**Results:**

Of 1376 men, the median age was 68 years, 51% were White, and 89% had bone-only metastases. Overall, 17%, 35%, and 25% of men received Ra-223 as first-line, second-line, or third-line treatment for mCRPC, respectively. Thirty-six percent received Ra-223 as combination/layered therapy, mainly with enzalutamide, and 46% completed ≥5 cycles. Overall, median rwOS was 22.9 months. Median rwOS was longer in men who completed ≥5 Ra-223 cycles versus 1–4 cycles (30.3 versus 15.3 months) and combination/layered therapy versus monotherapy (26.6 versus 20.5 months). Combination/layered therapy and completion of ≥5 Ra-223 cycles were associated with 22% and 55% reductions in risk of death in adjusted analyses, respectively. Limitations include some clinical information not captured by claims databases.

**Conclusions:**

Significant rwOS benefits were identified in men who received Ra-223 as an earlier line of therapy, received Ra-223 in combination with another therapy, and completed ≥5 Ra-223 cycles, underscoring the importance of Ra-223 in the current treatment landscape.

## Introduction

Radium-223 dichloride (Ra-223), an alpha-emitting radiopharmaceutical, has been an approved treatment for metastatic castration-resistant prostate cancer (mCRPC) with bone metastases and no known visceral involvement since 2013 [1]. Approval was based on the findings of the pivotal phase 3 ALSYMPCA trial where Ra-223 significantly prolonged overall survival (OS) and had quality of life benefits versus placebo [2, 3].

Since the approval of Ra-223, the treatment landscape for prostate cancer (PC) has evolved substantially. Changes include treatment intensification for metastatic hormone-sensitive prostate cancer (mHSPC), shifting from androgen deprivation therapy alone to in combination with androgen-receptor pathway inhibitors (ARPIs; i.e., enzalutamide, apalutamide, darolutamide, and abiraterone), with or without docetaxel [4–7], and the approval of ARPIs (enzalutamide, apalutamide, and darolutamide) for the treatment of non-metastatic castration-resistant prostate cancer (nmCRPC) [8–14]. As such, men with PC may receive more life-prolonging therapies prior to an mCRPC diagnosis.

Given the changes in PC treatment, recent real-world evidence of Ra-223 utilization patterns and outcomes may inform clinicians on when Ra-223 should be used and whether it is feasible to combine it with other agents. While numerous real-world studies of Ra-223 use in the United States (US) have been published, these are limited to only a few centers [15–18], have small sample sizes (*n* < 350) [15–21], or analyze data prior to the approval of ARPIs for nmCRPC/mHSPC or docetaxel for mHSPC [15, 16, 19, 21]. Consequently, existing data may not provide a complete picture of current Ra-223 treatment patterns and outcomes in the US. Therefore, there is a need for larger, more robust real-world studies evaluating Ra-223 use and associated clinical outcomes at a national level that integrate the current PC treatment paradigm. We aimed to comprehensively examine current treatment patterns and real-world OS (rwOS) in men with mCRPC treated with Ra-223 in clinical practice using data from a large US administrative claims dataset covering private health insurance.

## Subjects and methods

### Study design

We conducted a retrospective cohort analysis of men with PC who initiated Ra-223 (assumed, therefore, to be men with mCRPC) among private insurance beneficiaries, identified from claims made for Ra-223 from January 1, 2017 to June 30, 2022 (identification period). The index date was defined as the date of earliest Ra-223 administration; all individuals required a baseline period of ≥12 months prior to the index date and a follow-up period of ≥6 months, or until death if they died within 6 months from the index date. The end of follow-up was the last date of continuous enrollment, death, or the end of the study period (December 31, 2022). Study design details are shown in Supplementary Fig. S1.

### Ethics approval and consent to participate

This retrospective analysis of real-world data was conducted in accordance with the RECORD and STROBE statements [22, 23]. Due to the de-identified nature of the dataset, informed consent was not required; data were also considered exempt from Institutional Review Board review.

### Data source

The Komodo Health dataset is a verified, adjudicated, and de-identified administrative claims dataset covering >140 million individuals from >150 US private insurance providers. We utilized data from privately insured and Medicare Advantage Plan populations, representing individuals aged <65 years covered through employer-sponsored plans, health insurance exchange, or Medicaid managed care, as well as supplementary private insurance plans for individuals aged ≥65 years.

### Inclusion and exclusion criteria

We identified men with PC who received Ra-223 during the identification period using the selection process detailed in Supplementary Table S1.

### Ra-223 utilization patterns

We examined the completion of ≥5 Ra-223 cycles, the use of combination or layered therapy, and the sequence of PC therapies pre- and post-Ra-223. Ra-223 therapy completion was defined as completing ≥5 cycles without a gap of ≥56 days between two subsequent administrations. Combination therapy was considered the administration of other life-prolonging mCRPC medications within 30 days of starting Ra-223, and layered therapy was considered as starting Ra-223 after using another therapy for ≥30 days; these definitions are consistent with those of other studies [17, 24, 25].

The index line of therapy (LOT) was the use of Ra-223, as monotherapy or with any other mCRPC agent within 30 days of the index date. Change in LOT was defined as initiating a new PC non-index therapy or having a ≥90-day gap in the current LOT. Using these parameters, we also identified pre- and post-index LOTs during the baseline and follow-up periods, respectively.

### Real-world overall survival

The Komodo Health dataset contains mortality data from several sources, including claims or other mortality data sources, with 92% matching the death data reported by the Centers for Disease Control and Prevention in the year following 2017 (Komodo Health in-house resources). RwOS was calculated as the time from index date to date of death. Men whose follow-up end date was not due to death were censored at the end of follow-up.

### Study variables

Demographics at the index date, clinical characteristics, and PC treatments during the baseline period were analyzed; these covariates are defined in Supplementary Table S2.

### Statistical analyses

Descriptive statistics are reported for independent measures by overall population and study groups of interest. Logistic regression analyzed factors associated with completion of ≥5 Ra-223 cycles, including demographic, clinical, and medication-related characteristics. Odds ratios (ORs) were calculated to indicate baseline characteristics associated with completion of ≥5 Ra-223 cycles versus 1–4 cycles. Statistically significant differences in baseline characteristics between the Ra-223 monotherapy and combination/layered therapy groups were estimated using the chi-square or Fisher-exact tests for categorical variables and t-tests, Mann–Whitney or ANOVA tests for continuous variables. Median rwOS was calculated using Kaplan–Meier methodology. Cox-regression analyses examined associations between Ra-223 use and rwOS with adjustment for covariates, such as baseline demographics, clinical characteristics, treatments, and healthcare resource use.

## Results

### Patient demographics and baseline characteristics

During the sample identification process (Supplementary Table S1), of 556,610 men with metastatic prostate cancer, 11,069 (2%) had evidence of Ra-223 treatment initiation between 2017 to 2022. After applying further inclusion and exclusion criteria, 1376 men with mCRPC were included in this analysis (Supplementary Table S1). Demographic and baseline characteristics are summarized in Table 1. The median age was 68 years and 51% of men were White. On average, it took 2 years from the first observed metastasis or ADT use to initiate Ra-223. The majority of men (89%) had bone-only metastases and a Charlson Comorbidity Index (CCI) score ≥1 (76%). The most common pre-existing comorbidities were diabetes (34%), peripheral vascular disease (30%), pulmonary disease (26%), mild liver disease (25%), renal disease (20%), cerebral vascular accident (15%), and congestive heart failure (14%). Overall, 73%, 32%, 83%, and 74% of men had received prior ARPIs, chemotherapy, opioids and bone health agents (BHAs), respectively (Table 1).

**Table 1:** Demographics, baseline characteristics and treatment profiles of the overall study cohort (*N* = 1376).

Characteristic		Overall study cohort, *N* (%)	Characteristic		Overall study cohort, *N* (%)
Continuous age (years)^a^		68.7 [9.45]; 68 [61, 76]	CCI comorbidities	Diabetes	469 (34)
Time from first observed metastasis (in years)^a^		1.9 [1.2]; 1.7 [1.1, 2.6]		Peripheral vascular disease	412 (30)
Time from first observed ADT use (in years)^a^		2.0 [1.1]; 1.7 [1.2, 2.6]		Pulmonary disease	356 (26)
Age groups, years	18–64	573 (42)		Mild liver disease	346 (25)
	65–74	379 (28)		Renal disease	272 (20)
	75–79	188 (14)		Cerebral vascular accident	204 (15)
	80–84	187 (14)		Congestive heart failure	193 (14)
	≥85	49 (4)		Acute myocardial infarction	112 (8)
Race	White	697 (51)		Connective tissue disorder	46 (3)
	Black	221 (16)	Hospitalizations and hospital visits	Any inpatient hospitalization^b^	227 (17)
	Other	208 (15)		Any ED visit^b^	573 (42)
	Unknown	250 (18)		Number of outpatient visits,^b^ median [IQR]	27 [15, 42]
US census region	Midwest	321 (23)	Ra-223 index therapy	Combination	113 (8)
	Northeast	413 (30)		Layered	379 (28)
	South	407 (30)		Monotherapy	884 (64)
	West	235 (17)	Ra-223 index LOT^c^	1	240 (17)
Medical insurance type	Private/Medicaid	610 (44)		2	480 (35)
	Medicare Advantage	766 (56)		3	349 (25)
Types of metastases	Bone only	1225 (89)		4+	307 (22)
	Bone + visceral	151 (11)	ARPI use	Any	998 (73)
CCI categories^d^	0	328 (24)		ARI (enza, apa, daro)	646 (47)
	1–2	517 (38)		Abi	657 (48)
	3–4	314 (23)	Chemotherapy	Any	440 (32)
	5+	217 (16)		Docetaxel	410 (30)
Physician specialty	Urologist	27 (2)		Cabazitaxel	133 (10)
	Medical oncologist	179 (13)	PARPi		15 (1)
	Radiation oncologist	546 (40)	Sipuleucel-T		189 (14)
	Nuclear medicine	115 (8)	Opioid use		1143 (83)
	Diagnostic radiologist	100 (7)	Bone health agents		1014 (74)

*Abi* abiraterone, *ADT* androgen deprivation therapy, *apa* apalutamide, *ARI* androgen receptor inhibitor, *ARPI* androgen receptor pathway inhibitor, *CCI* Charlson Comorbidity Index, *daro* darolutamide, *ED* emergency department, *enza* enzalutamide, *IQR* interquartile range, *LOT* line of therapy, *SD* standard deviation, *PARPi* poly(ADP-ribose) polymerase inhibitor, *US* United States.

^a^Reported as mean [SD]; median [IQR].

^b^Baseline healthcare resource use (inpatient, ED, and outpatient visits) were captured during 12-month baseline period only.

^c^Baseline period was a minimum of 12 months to capture prior LOT (mean duration of baseline period was 32.8 months [SD: 16.3]).

^d^CCI was calculated by excluding index condition score for prostate cancer and metastases.

### Ra-223 utilization patterns

#### Sequence of Ra-223 use

**Fig. 1:**
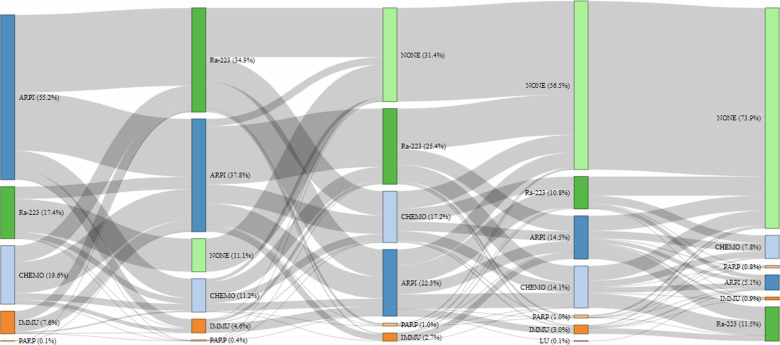
Treatment patterns from first- to fifth-line of therapy in men with prostate cancer. ARPI androgen receptor pathway inhibitor, CHEMO taxane-based chemotherapy, IMMU immunotherapy (sipuleucel-T or pembrolizumab), PARP poly (ADP-ribose) polymerase, LU Lutetium-177 vipivotide tetraxetan.

Ra-223 was most commonly received as a second (35%) or third (25%) LOT; 17% of men received Ra-223 as a first LOT (Fig. 1, Table 1). Few men received Ra-223 as a fourth (11%) or fifth (12%) LOT. For men who received Ra-223 as a second LOT, ARPIs (abiraterone [13%]; enzalutamide [12%]), docetaxel (5%), and sipuleucel-T (2%) were the most common first LOTs. When Ra-223 was given as a third LOT, back-to-back ARPIs (abiraterone-enzalutamide [5%]; enzalutamide-abiraterone [3%]) and docetaxel-ARPIs (docetaxel-abiraterone [3%]; docetaxel-enzalutamide [3%]) were common prior therapy sequences (Fig. 1). Post Ra-223 initiation, 45%, 19%, and 9% of men received one, two, or three subsequent LOTs, respectively, with chemo-ARPI and ARPI-chemo being the most common LOT sequences. The most common first subsequent LOTs were chemotherapy (docetaxel [31%]; cabazitaxel [15%]), ARPI (enzalutamide [22%]; abiraterone [21%]), and poly (ADP-ribose) polymerase inhibitor (olaparib [4%]). Similar trends were observed for second and third subsequent LOTs. Lutetium-177 vipivotide tetraxetan was used in <1% of subsequent LOTs.

#### Ra-223 monotherapy, layered, and combination therapy

**Fig. 2:**
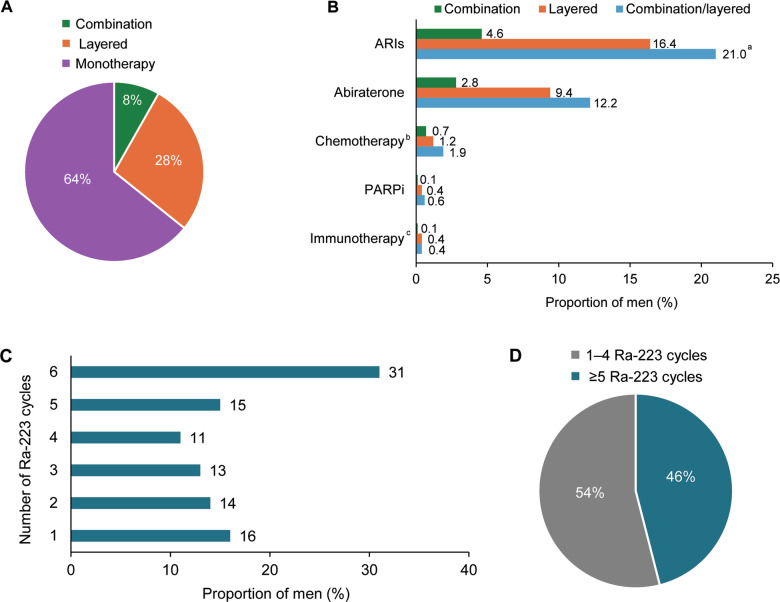
Combination therapy use and Ra-223 treatment completion. The proportion of men who **A** received Ra-223 as combination, layered or monotherapy, **B** received Ra-223 in combination/layered regimens with the specified agents, **C** completed the stated number of Ra-223 cycles, and **D** completed 1–4 or ≥5 Ra-223 cycles. ^a^Of the 289 men who received an ARI as combination/layered therapy, 275 (95%) received enzalutamide, 9 (3%) received apalutamide, and 5 (2%) received darolutamide. ^b^Taxane-based chemotherapy. ^c^Sipuleucel-T or pembrolizumab. ARI androgen receptor inhibitor, PARPi poly (ADP-ribose) polymerase inhibitor.

Ra-223 was given as monotherapy, layered, or combination therapy in 64%, 28%, and 8% of men, respectively (Fig. 2A; baseline characteristics for these groups are in Supplementary Table S3). ARIs (most commonly enzalutamide) and abiraterone were the most commonly used agents in combination/layered Ra-223 therapy (21% and 12%, respectively) (Fig. 2B).

In a multinomial logistic regression analysis, factors associated with a significantly higher likelihood of receiving layered Ra-223 therapy were pulmonary disease (OR 1.47; 95% confidence interval [CI] 1.07–2.03) and baseline BHA use (OR 1.47; 95% CI 1.08–1.99) (Supplementary Table S4). A lower likelihood of receiving layered Ra-223 therapy was associated with some older age groups (75–79 years [OR 0.58; 95% CI 0.33–0.99]; ≥85 years [OR 0.32; 95% CI 0.12–0.85]), a lower number of prior therapies (<2 [OR 0.58; 95% CI 0.42–0.80]) and pain medication use (OR 0.70; 95% CI 0.50–0.99). A higher likelihood of receiving Ra-223 combination therapy was associated with a lower number of prior therapies (<2 [OR 2.08; 95% CI 1.21–3.61]) and higher number of office visits (>17 [OR 1.58; 95% CI 1.03–2.42]) (Supplementary Table S4).

#### Completion of ≥5 Ra-223 cycles

Overall, 46% of men completed ≥5 Ra-223 cycles (Fig. 2C, D). In univariable analyses, men more likely to complete ≥5 Ra-223 cycles included those who had bone-only metastases, received Ra-223 as combination or layered therapy, received Ra-223 as first or second LOT, or had no prior abiraterone, chemotherapy or opioid use (Supplementary Table S5). In multivariable analyses, men who received Ra-223 as combination therapy had a higher likelihood of completing ≥5 Ra-223 cycles than those who received Ra-223 monotherapy (OR 1.59; 95% CI 1.01–2.50) (Supplementary Table S5).

### Real-world overall survival

**Fig. 3:**
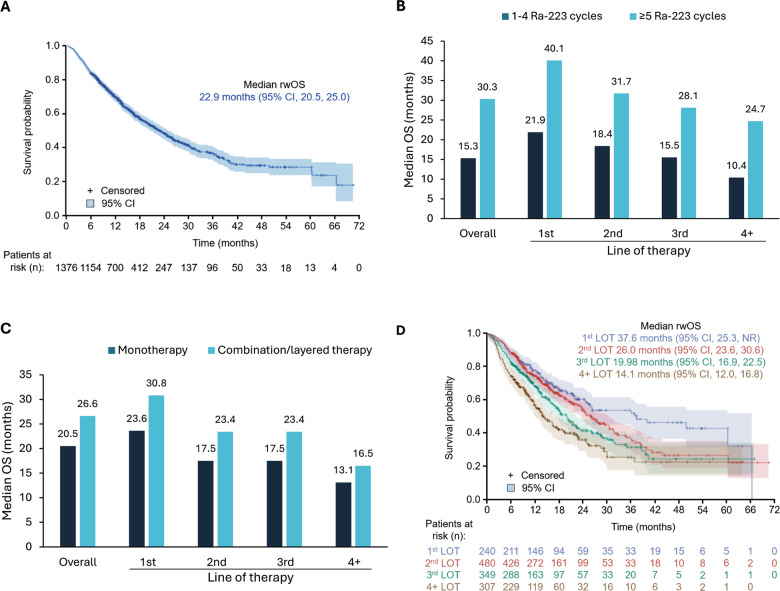
Real-world overall survival. Data are shown for **A** the overall cohort, **B** by completion of 1–4 versus ≥5 Ra-223 cycles and LOT, **C** by use of Ra-223 monotherapy versus combination/layered and LOT, and **D** by LOT. CI confidence interval, LOT line of therapy, rwOS real-world overall survival.

The median rwOS of the overall cohort was 22.9 months (Fig. 3A). Median rwOS was longer in men who completed ≥5 versus 1–4 Ra-223 cycles (30.3 versus 15.3 months) and in men who received Ra-223 as layered/combination therapy versus Ra-223 monotherapy (26.6 versus 20.5 months) (Table 2). Similar trends were seen irrespective of whether Ra-223 was the first, second, third or fourth LOT (Fig. 3B, C). In multivariable Cox-regression analyses, the risk of death was 55% lower in those completing ≥5 versus 1–4 Ra-223 cycles, and 22% lower when Ra-223 was used as combination/layered therapy versus monotherapy (Table 2, Supplementary Tables S6 and S7).

**Table 2:** Real-world overall survival in Ra-223 subgroups of interest.

Ra-223 subgroups	Median rwOS, months (95% CI)	Proportion of men who died (%)	Unadjusted HR (95% CI)	Adjusted HR^a^ (95% CI)
≥5 vs 1–4 Ra-223 cycles^b^	30.3 (26.4, 36.3) vs. 15.3 (13.2, 16.5)	36 vs. 53	0.43 (0.37–0.51)	0.45 (0.38–0.53)
Combination/layered vs. monotherapy^b^	26.6 (23.1, 30.9) vs. 20.5 (18.0, 23.7)	42 vs. 47	0.76 (0.64–0.90)	0.78 (0.65–0.93)
Line of therapy				
1 vs. 4+^b^	37.6 (25.3, NR) vs. 14.1 (12.0, 16.8)	38 vs. 53	0.47 (0.37–0.61)	0.42 (0.32–0.55)
2 vs. 4+^b^	26.0 (23.6, 30.6) vs. 14.1 (12.0, 16.8)	42 vs. 53	0.58 (0.47–0.71)	0.58 (0.47–0.72)
3 vs. 4+^b^	20.0 (16.9, 22.5) vs. 14.1 (12.0, 16.8)	47 vs. 53	0.73 (0.59–0.91)	0.75 (0.61–0.94)

*CI* confidence interval, *HR* hazard ratio, *NR* not reached, *rwOS* real-world overall survival.

^a^Independent characteristics comprised of baseline demographic characteristics (age, race, region), clinical conditions (Charlson Comorbidity Index, visceral metastases), baseline treatment use (line of therapy, pain medication), and baseline healthcare resource use (hospitalization, emergency room visit, median outpatient visits) in the adjusted model.

^b^All *P*-values were <0.05.

Median rwOS was longest in men who received Ra-223 as their first LOT (37.6 months) and decreased incrementally in subsequent LOTs (26.0, 20.0, and 14.1 months in second, third, or fourth or later LOT, respectively) (Fig. 3D). Compared with Ra-223 as fourth or later LOT, use as first, second, or third LOT was associated with a 58%, 42%, or 25% lower risk of death, respectively, when adjusted for other baseline variables (Table 2). In multivariable analysis, prior hospitalization and BHA use at baseline were associated with a higher risk of death; whereas African American race was associated with a lower risk of death (Supplementary Table S7).

## Discussion

Using a large US claims dataset of men with mCRPC treated with Ra-223 between January 2017 and June 2022, we describe how Ra-223 can impact survival. Our large cohort allowed us to analyze outcomes of numerous subgroups while maintaining sufficient sample sizes. Although Ra-223 is underutilized in the current mCRPC treatment landscape, our findings support its benefits as an established life-prolonging therapy.

Survival of the overall cohort was longer than in the ALSYMPCA trial (22.9 vs 14.9 months, respectively) [2], but remained within median ranges reported in other real-world studies (10.5–23.5 months [15–17, 20, 21, 26–36]). OS differences may be due to recent changes in the PC treatment landscape, such as the approval of ARPIs and chemotherapy for the treatment of nmCRPC and mHSPC [4–7]. Few life-prolonging therapies were available as standard treatments for mCRPC at the time of ALSYMPCA, although 57% of patients had received prior docetaxel; in a pre-specified subgroup analysis, Ra-223 prolonged OS versus placebo irrespective of prior docetaxel use [37]. In our study, 30% and 73% of men received prior docetaxel and ARPIs, respectively. Moreover, our study population was slightly younger than that of ALSYMPCA, due to high representation of younger (<65 years) private insurance beneficiaries, which may also have contributed to the prolonged rwOS observed in our study.

Real-world studies assessing if combining Ra-223 with other agents results in an observed improvement in survival versus Ra-223 monotherapy are limited and have not demonstrated significant findings [15, 19, 35]. However, these studies were limited by small cohorts (monotherapy and combination therapy groups: *N* = 128 and *N* = 92 [15]; *N* = 202 and *N* = 116 [19]; *N* = 32 and *N* = 19 [35]). By contrast, in our larger analysis, men in the combination/layered Ra-223 group (*N* = 492) had longer rwOS than those in the Ra-223 monotherapy group (*N* = 884). Among men who received combination/layered therapy, the agent most commonly given with Ra-223 was enzalutamide.

Several ongoing studies are assessing Ra-223 in combination with other mCRPC medications, including PEACE III (NCT02194842), DORA (NCT03574571), COMRADE (NCT03317392), and Rad2Nivo (NCT04109729). Notably, the phase 3 PEACE III trial recently demonstrated the significant clinical benefits of first-line combination therapy for mCRPC, with enzalutamide plus Ra-223 significantly prolonging both radiological progression-free survival (19.4 vs 16.4 months; *p* = 0.0009) and OS (42.3 vs 35.0 months; *p* = 0.0031) relative to enzalutamide alone [38]. Safety data from PEACE III indicate that concomitant BHA use mitigates the fracture risk of therapy, with the cumulative incidence of fractures at 1 year in men treated with enzalutamide plus Ra-223 being 2.7% (95% CI 0.5–8.5) with BHAs versus 37.1% (95% CI 21.3–53.0%) without BHAs; the corresponding values with enzalutamide were 2.6% (95% CI 0.5–8.3%) versus 15.6% (95% CI 5.6–30.3%) [39]. According to European [40, 41] and US [7] guidance, BHAs should be considered for all men with mCRPC [40] with bone metastases [7, 41] in order to prevent/delay skeletal-related events such as osteoporotic fragility fractures. Advanced prostate cancer carries an increased risk of osteoporosis [42] (which can be exacerbated by the use of ADT [40, 43]), with this poor bone health leading to bone fragility and fractures in some patients [44], including those treated with Ra-223 [45].

In our study, most men received Ra-223 after one or two LOT. Consistent with many drugs, Ra-223 may provide a greater survival benefit when provided as a first versus second or later LOT. Similar findings have been seen in other real-world studies [31, 46]. As patients progress through several lines of therapy, their general health can deteriorate (e.g., declining performance status, disease or symptomatic progression, or cytopenia), potentially leading to early treatment discontinuation. In our study, men who completed ≥5 Ra-223 cycles were observed to live longer than men who only completed 1–4 cycles, consistent with findings of other real-world studies [15, 27, 29]. We found that completing ≥5 Ra-223 cycles was more likely when Ra-223 was used as combination therapy than when used as layered therapy or monotherapy, supporting a previous real-world finding that men receive more cycles when Ra-223 is given as combination therapy versus monotherapy [15]. Prior studies have shown poor performance status and high tumor markers may be associated with lower ability to complete Ra-223 treatment [27, 47]. Due to data limitations, we were unable to capture such information. Nevertheless, our findings support the importance of identifying the population that will be able to adhere to the full treatment course and receive the optimal benefits of Ra-223 therapy. Currently, there are no validated markers for predicting which men with mCRPC are likely to respond the best to Ra-223, although several have shown promise including automated bone scan index, circulating tumor cells, alkaline phosphatase, lactate dehydrogenase, and bone metabolic markers [46, 48–50].

With respect to race, there was no significant difference in completion of ≥5 Ra-223 cycles between Whites and African Americans. Furthermore, with respect to survival, African Americans had a lower risk of death compared to Whites. Our findings are consistent with a large systematic literature review showing no difference or favorable outcomes for African American men versus White men with mCRPC when they were treated with specific therapies, including Ra-223, in real-world practice [51].

Despite the strengths of the study, it is not without limitations. First, while we were able to capture opioid and BHA use, we were not able to capture pain or disease severity with number of metastases and prostate-specific antigen or alkaline phosphatase levels. Second, it is possible that some of the prior LOTs for mCRPC could have been utilized for mHSPC, which might have led to a misclassification of second- and third-line Ra-223 LOT. However, when comparing these data with those of a chart review [21], we found relative consistency in the distribution of index Ra-223 LOTs. Therefore, the degree of misclassification for second- and third LOTs is likely negligible. Third, the findings may not be generalizable to public insurance-only beneficiaries. Fourth, we were unable to evaluate how approval of lutetium (^177^Lu) vipivotide tetraxetan (a beta-emitter) for men with mCRPC may have impacted Ra-223 treatment patterns, as its approval was 3 months prior to our data cutoff, and its usage was therefore low.

## Conclusions

To our knowledge, this is the largest analysis of a real-world cohort of men treated with Ra-223 in the US. Our analysis addresses the utilization of Ra-223 in the current mCRPC treatment landscape. Our findings support using Ra-223 as an earlier LOT, giving Ra-223 in combination with another life-prolonging therapy, and the importance of completing ≥5 cycles to improve survival outcomes. Real-world evidence can provide information pertaining to treatment patterns and associated survival outcomes in men with mCRPC, which may be used to guide clinical decisions.

## Supplementary information

Supplementary material

## Data Availability

Availability of the data underlying this publication will be determined according to Bayer’s commitment to the EFPIA/PhRMA “Principles for responsible clinical trial data sharing”. This pertains to scope, timepoint, and process of data access. As such, Bayer commits to sharing upon request from qualified scientific and medical researchers patient-level clinical trial data, study-level clinical trial data, and protocols from clinical trials in patients for medicines and indications approved in the United States (US) and European Union (EU) as necessary for conducting legitimate research. This applies to data on new medicines and indications that have been approved by the US and EU regulatory agencies on or after January 01, 2014. Interested researchers can use www.clinicalstudydatarequest.com to request access to anonymized patient-level data and supporting documents from clinical studies to conduct further research that can help advance medical science or improve patient care. Information on the Bayer criteria for listing studies and other relevant information is provided in the Study sponsors section of the portal. Data access will be granted to anonymized patient-level data, protocols, and clinical study reports after approval by an independent scientific review panel. Bayer is not involved in the decisions made by the independent review panel. Bayer will take all necessary measures to ensure that patient privacy is safeguarded.
