# Supplementary material for: Real-world utilization patterns and survival in men with metastatic prostate cancer treated with Radium-223 in the United States
Source: Prostate Cancer Prostatic Dis. 2025 Apr 4;28(4):960–7. doi: 10.1038/s41391-025-00969-6 (PMC12643915; doi:10.1038/s41391-025-00969-6)
Supplement: Supplementary file 1 — Supplementary material [file 41391_2025_969_MOESM1_ESM.docx]

Supplementary tables and figures

**Table S1. Study cohort identification and selection process**

| **Steps** | **Criteria** | **N** |
| --- | --- | --- |
| 1 | ≥2 medical claims with diagnosis codes for prostate cancer on distinct dates between 01/01/2016 to 12/31/2022 | 3 120 556 |
| 2 | ≥1 medical claim with diagnosis codes for metastases on/after prostate cancer date the index date | 556 610 |
| 3 | ≥1 medical/pharmacy claim(s) with the procedure codes for Ra-223 during 01/01/2017 to 06/30/2022^a^ The earliest date of Ra-223 use was assigned as the index date | 11 069 |
| 4 | No medical claims with diagnosis codes for other cancers (except for prostate and skin cancer), prior to the earliest claim of prostate cancer | 10 277 |
| 5 | ≥12 months of continuous enrollment with medical and pharmacy benefits prior to the index date^b^ | 1 747 |
| 6 | ≥6 months of post-index continuous enrollment, or death if died within 6 months post-index | 1 471 |
| 7 | No prior medical or pharmacy claim with a procedure code for Ra-223 during the baseline period | 1 377 |
| 8 | Men with bone metastases | 1,376 |
| 9 | Men aged ≥18 years at the index date | 1 376 |

^a^Ra-223 use was identified based on claims with related Healthcare Common Procedure Coding System or National Drug Code; pharmacy claims flagged as “PAID” were included in analysis.

^b^A 45-day gap between adjacent payer spans in enrollment file was allowed.

**Table S2. Baseline demographic and clinical characteristics**

| **Variable** | **Definition** | **Reporting metric** |
| --- | --- | --- |
| **Demographic characteristics on the index date** | | |
| Age | Age (years) was reported as a continuous variable and categorical variable with the following categories:   - 18–64 - 65–74 - 75–79 - 80–84 - ≥85 | Mean (SD)/Median (IQR) for the continuous age variable  N (%) for the categorical age variable |
| US geographic region | Region was classified as:   - North central - South - West - Northeast | N (%) |
| Insurance type | Payer type was classified as:   - Private - Medicare Advantage | N (%) |
| Physician specialty | Type of physician specialty at Ra-223 initiation on the index date was identified using medical claims closest to the initiation of Ra-223. We reported the following specialties:   - Urologist - Oncologist/Radiation oncologist - Nuclear medicine - Diagnostic radiologist - Other | N (%) |
| Year of Ra-223 initiation | Year of the initiation of Ra-223 was reported as:   - 2017 - 2018 - 2019 - 2020 - 2021 - 2022 | N (%) |
| **Clinical characteristics in the baseline period** | | |
| CCI^a^ | The presence of medical conditions was identified using medical claims; we confirmed the presence or absence of each of the conditions listed in the CCI during the baseline period as below:   - Acute myocardial infarction (1) - Congestive heart failure (1) - Peripheral vascular disease (1) - Cerebral vascular accident (1) - Dementia (1) - Pulmonary disease (1) - Connective tissue disorder (1) - Peptic ulcer (1) - Mild liver disease (1) - Diabetes without complications (1) - Diabetes with complications (2) - Hemiplegia or paraplegia (2) - Renal disease (2) - Cancer (2) - Severe liver disease (3) - Metastatic solid tumors (6) - AIDS/HIV (6)   The CCI was calculated for each man based on all diagnoses during the baseline period and the index date; the following variables were reported:   - CCI - CCI category: - 0 - 1–2 - 3–4 - 5–6 - ≥7 | Mean (SD)/Median (IQR) for the continuous measure  N (%) for the categorical measure |
| Types of metastases | The presence of any type of metastases was confirmed using diagnosis codes for different metastasis types in the medical claims during the baseline period  The proportions of men with the following types of metastases during the baseline period (including the index date) were identified:   - Bone metastases only - − lymph node - + lymph node - Bone + visceral or other metastases - Visceral metastases in the absence of documented bone metastases | N (%) |
| **Prostate cancer treatment in the baseline period** | | |
| Baseline LOT | The earliest use of prostate cancer medication and use of any other agent within 30 days of the earliest use as the earliest LOT, prior to the index date    Changes in the LOT were defined as the addition or use of medication not part of the preceding regimen, or a gap of 90 days within the preceding regimen | N (%) |
| Radiation | ≥1 medical procedure code(s) for radiation therapy within medical claims during the baseline period | N (%) |
| Surgery | ≥1 medical procedure code(s) for surgery within medical claims during the baseline period. We reported the use of the following surgical procedures:   - Prostatectomy - Cryosurgery | N (%) |
| Surgical castration | ≥1 medical procedure code(s) for orchiectomy within medical claims during the baseline period | N (%) |
| ADT/medical castration therapy | ≥1 HCPCS code(s) within medical claims, or ≥1 NDC(s) within prescription claims for ADT agents during the baseline period. We reported a composite unit as use of any or individual ADT agent, as below:   - Any of the following ADT: - LHRH agonist - Goserelin - Histrelin - Leuprolide - Triptorelin - LHRH antagonist - Degarelix - Relugolix | N (%) |
| FGARI | ≥1 HCPCS/CPT code(s) within medical claims, or ≥1 NDC code(s) within prescription claims for FGARIs during the baseline period. We reported a composite unit as use of any or individual FGARI agents, as below:   - Any FGARI - Bicalutamide - Flutamide - Nilutamide | N (%) |
| SGARI | ≥1 NDC code(s) for any of the SGARIs within prescription claims during the baseline period. We reported a composite unit as the use of any or individual SGARI agent, as below:   - Any SGARI - Enzalutamide - Apalutamide - Darolutamide | N (%) |
| AR biosynthesis inhibitors | ≥1 NDC code(s) within prescription claims for abiraterone during the baseline period | N (%) |
| Chemotherapy | ≥1 HCPCS/CPT code(s) within medical claims for chemotherapy, or ≥1 NDC code(s) for chemotherapy within prescription claims during the baseline period. We report a composite unit as use of any or individual chemotherapy agents:   - Chemotherapy   - Docetaxel   - Cabazitaxel | N (%) |
| Immunotherapy | ≥1 HCPCS/CPT code(s) for administration of any of the agents utilized for immunotherapy within medical claims or ≥1 prescription fill(s) for immunotherapy within prescription claims during the baseline period:   - Pembrolizumab - Sipuleucel-T | N (%) |
| Lutetium-177 vipivotide tetraxetan | ≥1 HCPCS/CPT code(s) within medical claims or ≥1 prescription fill(s) with NDC code(s) within prescription claims for lutetium-177 vipivotide tetraxetan during the baseline period | N (%) |
| PARPi | ≥1 prescription fill(s) with NDC code(s) for PARPi(s) within prescription claims during the baseline period:   - Olaparib - Rucaparib | N (%) |
| Baseline pain medication | ≥1 NDC code(s) within prescription claim(s) for opioid analgesics during the baseline period | N (%) |
| Baseline BHA | ≥1 NDC code(s) within prescription claims for BHA(s) during the baseline period:   - Denosumab - Zoledronic acid - Etidronate - Ibandronate - Pamidronate - Risedronate - Alendronate | N (%) |
| **Healthcare resource utilization in the baseline period** | | |
| Healthcare resource utilization | The proportion of men with utilization of healthcare services and the number of visit types were reported. All-cause resource utilization included use (for any reason) of medical and pharmacy services. Each of the following were measured and reported:   - Hospitalization: a binary indicator of ≥1 hospitalization and a continuous measure of the total number of hospitalizations and total length of stay across hospitalizations - ED visits: a binary indicator for ≥1 ED visit and a continuous measure of the total number of ED visits per patient - Outpatient visits: a binary indicator of ≥1 outpatient visit and a continuous measure of the total number of outpatient visits per patient - Pharmacy: a binary indicator of ≥1 pharmacy refill(s) and a continuous measure of the total number of distinct medication fills per patient | N (%)  Mean (SD)/Median (IQR) |

^a^The CCI is a method of estimating 1-year mortality for men with certain comorbid conditions. Each comorbid condition is given a score of 1, 2, 3, or 6 based on the degree of mortality attributed to the condition (Glasheen WP et al. Am Health Drug Benefits. 2019;12(4):188-197). Individuals with metastatic solid tumors are assigned a score of 6 and any malignancy a score of 2. Since the study population comprised men with metastatic prostate cancer, we deducted the CCI score for malignancy and metastases in this study to reflect the burden of other clinical conditions.

ADT, androgen deprivation therapy; AR, androgen receptor; BHA, bone health agent; CCI, Charlson Comorbidity Index; ED, emergency department; FGARI, first-generation androgen receptor inhibitor; HCPCS/CPT, healthcare procedure coding system/current procedural terminology; IQR, interquartile range; LHRH, luteinizing hormone releasing hormone; LOT, line of therapy; PARPi, poly (ADP-ribose) polymerase inhibitor; NDC, national drug code; SD, standard deviation; SGARI, second-generation androgen receptor inhibitor; US, United States.

**Table S3. Baseline characteristics of men in the Ra-223 monotherapy, layered, and combination therapy cohorts**

| **Characteristics** | **Monotherapy**  **N = 884 (%)** | **Layered**  **N = 379 (%)** | **Combination**  **N = 113 (%)** | ***P* value^a^** |
| --- | --- | --- | --- | --- |
| Mean continuous age [SD]; median [IQR] | 69.6 [9.39];  70 [62, 77] | 67.1 [9.25];  65 [61, 74] | 66.7 [9.62];  64 [59, 75] | <0.001 |
| Age groups, years |  |  |  | 0.001 |
| 18–64 | 330 (37) | 185 (49) | 58 (51) |  |
| 65–74 | 254 (29) | 100 (26) | 25 (22) |  |
| 75–79 | 132 (15) | 43 (11) | 13 (12) |  |
| 80–84 | 126 (14) | 45 (12) | 16 (14) |  |
| ≥85 | 42 (5) | 6 (2) | 1 (1) |  |
| Race |  |  |  | 0.021 |
| White | 475 (54) | 171 (45) | 51 (45) |  |
| Black | 142 (16) | 64 (17) | 15 (13) |  |
| Other | 115 (13) | 68 (18) | 25 (22) |  |
| Unknown | 152 (17) | 76 (20) | 22 (19) |  |
| Region |  |  |  | 0.002 |
| Midwest | 237 (27) | 68 (18) | 16 (14) |  |
| Northeast | 249 (28) | 131 (35) | 33 (29) |  |
| South | 258 (29) | 111 (29) | 38 (34) |  |
| West | 140 (16) | 69 (18) | 26 (23) |  |
| Medical insurance type |  |  |  | <0.001 |
| Private/Medicaid | 354 (40) | 192 (51) | 64 (57) |  |
| Medicare Advantage | 530 (60) | 187 (49) | 49 (43) |  |
| Ra-223 treatment insurance type |  |  |  | <0.001 |
| Private/Medicaid | 359 (41) | 195 (51) | 64 (57) |  |
| Medicare Advantage | 525 (59) | 184 (49) | 49 (43) |  |
| Year of Ra-223 initiation |  |  |  | 0.011 |
| 2017 | 186 (21) | 114 (30) | 40 (35) |  |
| 2018 | 206 (23) | 82 (22) | 25 (22) |  |
| 2019 | 170 (19) | 60 (16) | 20 (18) |  |
| 2020 | 141 (16) | 58 (15) | 14 (12) |  |
| 2021 | 134 (15) | 45 (12) | 12 (11) |  |
| 2022 (up to 30^th^ June) | 47 (5) | 20 (5) | 2 (2) |  |
| Physician specialty |  |  |  |  |
| Urologist | 22 (2) | 5 (1) | 0 (0) | 0.114 |
| Radiation oncologist | 323 (37) | 169 (45) | 54 (48) | 0.005 |
| Medical oncologist | 126 (14) | 43 (11) | 10 (9) | 0.145 |
| Nuclear medicine | 80 (9) | 28 (7) | 7 (6) | 0.426 |
| Diagnostic radiologist | 59 (7) | 32 (8) | 9 (8) | 0.517 |
| Other/missing | 353 (40) | 126 (33) | 40 (35) | 0.07 |
| Types of metastases |  |  |  | 0.991 |
| Bone only | 787 (89) | 337 (89) | 101 (89) |  |
| Bone + visceral | 97 (11) | 42 (11) | 12 (11) |  |
| CCI categories |  |  |  | 0.180 |
| 0 | 205 (23) | 100 (26) | 23 (20) |  |
| 1–2 | 324 (37) | 146 (39) | 47 (42) |  |
| 3–4 | 207 (23) | 75 (20) | 32 (28) |  |
| 5+ | 148 (17) | 58 (15) | 11 (10) |  |
| CCI comorbidities |  |  |  |  |
| Any diabetes | 297 (34) | 127 (34) | 45 (40) | 0.405 |
| Diabetes without complications | 289 (33) | 126 (33) | 45 (40) | 0.317 |
| Diabetes with complications | 155 (18) | 60 (16) | 21 (19) | 0.698 |
| Peripheral vascular disease | 282 (32) | 100 (26) | 30 (27) | 0.104 |
| Pulmonary disease | 225 (25) | 110 (29) | 21 (19) | 0.075 |
| Mild liver disease | 228 (26) | 86 (23) | 32 (28) | 0.365 |
| Renal disease | 179 (20) | 74 (20) | 19 (17) | 0.682 |
| Cerebral vascular accident | 150 (17) | 41 (11) | 13 (12) | 0.011 |
| Congestive heart failure | 140 (16) | 44 (12) | 9 (8) | 0.021 |
| ARIs |  |  |  |  |
| Any ARI | 344 (39) | 278 (73) | 24 (21) | <0.0001 |
| Enzalutamide | 322 (38) | 273 (72) | 23 (20) | <0.0001 |
| Apalutamide | 18 (2) | 8 (2) | 2 (2) | 0.975 |
| Darolutamide | 4 (0.5) | 1 (0.3) | 0 (0) | 0.701 |
| AR biosynthesis inhibitors |  |  |  |  |
| Abiraterone | 412 (47) | 208 (55) | 37 (33) | <0.0001 |
| Any ARI or abiraterone | 568 (64) | 372 (98) | 58 (51) | <0.0001 |
| Chemotherapy |  |  |  |  |
| Any chemotherapy | 313 (35) | 97 (26) | 30 (27) | 0.001 |
| Docetaxel | 289 (33) | 92 (24) | 29 (26) | 0.007 |
| Cabazitaxel | 110 (12) | 18 (5) | 5 (4) | <0.0001 |
| Immunotherapy |  |  |  |  |
| Any immunotherapy | 106 (12) | 73 (19) | 19 (17) | 0.003 |
| Pembrolizumab | 8 (1) | 3 (1) | 1 (1) | 0.980 |
| Sipuleucel-T | 99 (11) | 72 (19) | 18 (16) | 0.001 |
| Lutetium-177 vipivotide tetraxetan | 0 (0) | 0 (0) | 0 (0) | 0 |
| PARPi |  |  |  |  |
| Any PARPi | 7 (1) | 8 (2) | 0 (0) | 0.060 |
| Rucaparib | 1 (<1) | 0 (0) | 0 (0) | 0.757 |
| Olaparib | 7 (1) | 8 (2) | 0 (0) | 0.060 |
| Pain medication |  |  |  |  |
| Any pain medication | 741 (84) | 304 (80) | 98 (87) | 0.163 |
| BHA |  |  |  |  |
| Any BHA | 628 (71) | 302 (80) | 84 (74) | 0.006 |
| Prior LOT |  |  |  | 0.001 |
| 0 | 199 (23) | 0 (0) | 41 (36) |  |
| 1 | 291 (33) | 153 (40) | 36 (32) |  |
| 2 | 202 (23) | 130 (34) | 17 (15) |  |
| 3 | 99 (11) | 41 (11) | 9 (8) |  |
| 4+ | 93 (11) | 55 (15) | 10 (9) |  |
| Healthcare resource utilization |  |  |  |  |
| ≥1 hospitalization | 169 (19) | 58 (15) | 24 (21) | 0.189 |
| ≥1 ED visit | 430 (49) | 156 (41) | 56 (50) | 0.041 |
| Median number of office visits [IQR] | 16 [6, 27] | 19 [9, 29] | 21 [10, 34] | <0.001 |
| Median number of  out-patient visits [IQR] | 29 [16, 43] | 24 [14, 41] | 25 [14, 37] | 0.097 |

^a^ANOVA tests were conducted for continuous variables.

AR, androgen receptor; ARI, androgen receptor inhibitor; BHA, bone health agent; CCI, Charlson Comorbidity Index; ED, emergency department; IQR, interquartile range; LOT, line of therapy; PARPi, poly(ADP-ribose) polymerase inhibitor; SD, standard deviation.

**Table S4. Multinomial logistic regression analysis examining the factors associated with the use of Ra-223 layered and combination therapy**

| Characteristics | Layered | | Combination | |
| --- | --- | --- | --- | --- |
|  | OR  (95% CI) | *P* value | OR  (95% CI) | *P* value |
| Age groups (Ref = 18–64) |  |  |  |  |
| 65–74 | 0.73 (0.47–1.12) | 0.149 | 1.03 (0.51–2.07) | 0.939 |
| 75–79 | 0.58 (0.33–0.99) | 0.046 | 1.24 (0.52–2.95) | 0.624 |
| 80–84 | 0.62 (0.36–1.09) | 0.095 | 1.69 (0.71–4.00) | 0.237 |
| ≥85 | 0.32 (0.12–0.85) | 0.022 | 0.36 (0.04–3.04) | 0.351 |
| Race (Ref = White) |  |  |  |  |
| Black | 1.26 (0.88–1.81) | 0.204 | 0.97 (0.52–1.82) | 0.928 |
| Other | 1.34 (0.92–1.94) | 0.123 | 1.33 (0.76–2.32) | 0.317 |
| Unknown | 1.08 (0.74–1.57) | 0.705 | 0.90 (0.50–1.63) | 0.734 |
| Region (Ref = West) |  |  |  |  |
| Midwest | 0.70 (0.46–1.07) | 0.097 | 0.51 (0.26–1.02) | 0.056 |
| Northeast | 1.27 (0.87–1.85) | 0.222 | 0.94 (0.52–1.68) | 0.822 |
| South | 0.94 (0.64–1.37) | 0.751 | 0.83 (0.47–1.45) | 0.509 |
| Medical insurance type (Ref = Other) |  |  |  |  |
| Private/Medicaid | 0.99 (0.64–1.54) | 0.959 | 1.79 (0.89–3.63) | 0.104 |
| Physician specialty (Ref = Urologist) |  |  |  |  |
| Radiation/Medical Oncologists | 1.16 (0.90–1.50) | 0.262 | 1.05 (0.70–1.59) | 0.805 |
| CCI |  |  |  |  |
| CCI Categories (Ref = 5+) |  |  |  |  |
| 0 | 0.87 (0.42–1.80) | 0.714 | 0.96 (0.29–3.18) | 0.940 |
| 1–2 | 0.91 (0.52–1.59) | 0.738 | 1.36 (0.53–3.47) | 0.518 |
| 3–4 | 0.76 (0.47–1.22) | 0.253 | 1.68 (0.74–3.82) | 0.212 |
| CCI Comorbidities (score in CCI) |  |  |  |  |
| Diabetes (Ref = No) | 1.04 (0.74–1.46) | 0.822 | 1.33 (0.80–2.22) | 0.269 |
| Peripheral vascular disease  (Ref = No) | 0.86 (0.62–1.18) | 0.344 | 0.94 (0.57–1.56) | 0.806 |
| Mild liver disease (Ref = No) | 0.83 (0.60–1.15) | 0.268 | 1.20 (0.72–2.00) | 0.486 |
| Pulmonary disease (Ref = No) | 1.47 (1.07–2.03) | 0.018 | 0.59 (0.34–1.03) | 0.065 |
| Congestive heart failure  (Ref = No) | 0.88 (0.57–1.35) | 0.545 | 0.67 (0.31–1.47) | 0.317 |
| Cerebral vascular accident  (Ref = No) | 0.78 (0.51–1.19) | 0.245 | 0.71 (0.36–1.39) | 0.314 |
| Acute myocardial infarction  (Ref = No) | 1.43 (0.84–2.41) | 0.185 | 0.65 (0.24–1.78) | 0.403 |
| Types of metastases |  |  |  |  |
| Bone + visceral (Ref = Bone only) | 1.09 (0.73–1.63) | 0.670 | 0.94 (0.49–1.81) | 0.845 |
| Number of prior therapies (Ref = 3+) |  |  |  |  |
| <2 | 0.58 (0.42–0.80) | 0.001 | 2.08 (1.21–3.61) | 0.009 |
| 2 | 1.33 (0.95–1.87) | 0.096 | 0.77 (0.39–1.54) | 0.461 |
| Pain medication (Ref = No) | 0.70 (0.50–0.99) | 0.045 | 1.42 (0.78–2.60) | 0.258 |
| BHAs (Ref = No) | 1.47 (1.08–1.99) | 0.013 | 1.04 (0.65–1.66) | 0.887 |
| Baseline healthcare resource utilization |  |  |  |  |
| Any hospitalization (Ref = No) | 0.92 (0.63–1.34) | 0.661 | 1.34 (0.77–2.34) | 0.293 |
| Any ED visit (Ref = No) | 0.82 (0.62–1.08) | 0.156 | 1.15 (0.74–1.80) | 0.532 |
| Median office visits >17 (Ref = ≤17 visits) | 1.24 (0.95–1.61) | 0.108 | 1.58 (1.03–2.42) | 0.037 |
| Median outpatient visits >27 (Ref = ≤27 visits) | 0.79 (0.60–1.05) | 0.099 | 0.81 (0.51–1.27) | 0.352 |

BHA, bone health agent; CI, confidence interval; CCI, Charlson Comorbidity Index; ED, emergency department; OR, odds ratio.

**Table S5. Univariable and multivariable regression analysis of factors associated with the completion of ≥5 Ra-223 cycles in the overall cohort**

| **Baseline characteristics** | **Univariable** | | **Multivariable** | |
| --- | --- | --- | --- | --- |
|  | **N (%)^a^** | ***P* value** | **OR (95% CI)** | ***P* value** |
| Sample size | 638 (46) |  |  |  |
| Continuous age, mean [SD]/median [IQR] | 69.0 [9.53]/  69 [61, 77] | 0.291 |  |  |
| Age group |  | 0.514 |  |  |
| 18–64 | 266 (46) |  | Ref |  |
| 65–74 | 163 (43) |  | 0.97 (0.63–1.47) | 0.870 |
| 75–79 | 93 (50) |  | 1.30 (0.78–2.18) | 0.316 |
| 80–84 | 91 (49) |  | 1.13 (0.67–1.93) | 0.642 |
| ≥85 | 25 (51) |  | 1.40 (0.65–3.00) | 0.394 |
| Race |  | 0.395 |  |  |
| White | 320 (46) |  | Ref |  |
| Black | 106 (48) |  | 1.00 (0.71–1.42) | 0.992 |
| Other | 105 (50) |  | 1.11 (0.77–1.59) | 0.576 |
| Unknown | 107 (43) |  | 0.94 (0.65–1.35) | 0.724 |
| US census region |  | 0.238 |  |  |
| Midwest | 147 (46) |  | 1.23 (0.83–1.80) | 0.302 |
| Northeast | 208 (50) |  | 1.32 (0.91–1.91) | 0.142 |
| South | 182 (45) |  | 0.96 (0.67–1.39) | 0.841 |
| West | 101 (43) |  | Ref |  |
| Medical insurance type |  | 0.814 |  |  |
| Private/Medicaid | 285 (47) |  | 1.04 (0.69–1.57) | 0.851 |
| Medicare | 353 (46) |  | Ref |  |
| Physician specialty |  |  |  |  |
| Urologist | 14 (52) | 0.564 | Ref |  |
| Medical oncologist | 76 (43) | 0.261 | 0.64 (0.25–1.64) | 0.356 |
| Radiation oncologist | 289 (53) | <0.001 | 0.87 (0.36–2.11) | 0.760 |
| Nuclear medicine | 47 (41) | 0.217 | 0.60 (0.23–1.59) | 0.308 |
| Diagnostic radiologist | 45 (45) | 0.776 | 0.68 (0.25–1.80) | 0.435 |
| Others | 219 (42) | 0.016 | 0.57 (0.23–1.39) | 0.216 |
| Types of metastases |  | 0.007 |  |  |
| Bone only | 353 (50) |  | Ref |  |
| Bone + visceral | 285 (43) |  | 0.89 (0.69–1.14) | 0.346 |
| CCI |  |  |  |  |
| CCI categories |  | 0.474 |  |  |
| 0 | 142 (43) |  | 0.37 (0.19–0.75) | 0.005 |
| 1–2 | 251 (49) |  | 0.68 (0.40–1.15) | 0.146 |
| 3–4 | 142 (45) |  | 0.71 (0.45–1.12) | 0.139 |
| 5+ | 103 (48) |  | Ref |  |
| CCI comorbidities (>10%; ref = no) |  |  |  |  |
| Diabetes | 226 (48) | 0.330 | 0.77 (0.56–1.06) | 0.105 |
| Peripheral vascular disease | 183 (44) | 0.343 | 0.78 (0.58–1.06) | 0.107 |
| Pulmonary disease | 158 (44) | 0.383 | 0.74 (0.54–1.01) | 0.055 |
| Mild liver disease | 163 (47) | 0.749 | 1.11 (0.81–1.52) | 0.503 |
| Renal disease | 135 (50) | 0.228 | 1.09 (0.67–1.80) | 0.723 |
| Cerebral vascular accident | 87 (43) | 0.248 | 0.72 (0.49–1.05) | 0.090 |
| Congestive heart failure | 84 (44) | 0.393 | 0.89 (0.60–1.33) | 0.566 |
| Ra-223 index therapy |  | 0.003 |  |  |
| Combination | 63 (56) |  | 1.59 (1.01–2.50) | 0.047 |
| Layered | 194 (51) |  | 1.17 (0.87–1.57) | 0.301 |
| Monotherapy | 381 (43) |  | Ref |  |
| LOT |  | 0.001 |  |  |
| 0 | 121 (50) |  | 0.63 (0.25–1.64) | 0.347 |
| 1 | 242 (50) |  | 0.95 (0.51–1.75) | 0.860 |
| 2 | 164 (47) |  | 1.20 (0.78–1.85) | 0.397 |
| 3 | 49 (33) |  | Ref = 3+ |  |
| 4+ | 62 (39) |  |  |  |
| ARI (Ref = no) | 291 (45) | 0.356 | 0.74 (0.49–1.11) | 0.143 |
| Abiraterone (Ref = no) | 270 (41) | <0.001 | 0.64 (0.43–0.94) | 0.025 |
| ARPI use (Ref = no) | 444 (45) | 0.023 | 0.69 (0.45–1.05) | 0.084 |
| Chemotherapy (Ref = no) | 165 (38) | <0.001 | 1.25 (0.80–1.95) | 0.321 |
| PARPi (Ref = no) | 5 (33) | 0.309 |  |  |
| Opioid use (Ref = no) | 510 (45) | 0.004 | 0.77 (0.56–1.08) | 0.129 |
| BHA (Ref = no) | 468 (46) | 0.791 | 0.92 (0.70–1.22) | 0.566 |
| Any hospitalization (Ref = no) | 103 (41) | 0.061 | 1.06 (0.74–1.51) | 0.755 |
| Any ED visit (Ref = no) | 277 (43) | 0.025 | 1.01 (0.77–1.32) | 0.957 |
| Office visits >17  (Ref = ≤17 visits) | 597 (47) | 0.705 | 1.22 (0.94–1.56) | 0.130 |
| Outpatient visits >27  (Ref = ≤27 visits) | 633 (46) | 0.069 | 0.79 (0.60–1.03) | 0.076 |
| Shorter survival |  |  |  |  |
| Survived <6 months   (Ref = survived ≥6 months) | 13 (6) | <0.01 | 0.05 (0.03–0.09) | <0.0001 |

^a^ Row % values were calculated from the numbers presented in the equivalent rows in Table 1.

ARI, androgen receptor inhibitor; ARPI, androgen receptor pathway inhibitor; BHA, bone health agent; CI, confidence interval; CCI, Charlson Comorbidity Index; ED, emergency department; IQR, interquartile range; LOT, line of therapy; OR, odds ratio; PARPi, poly (ADP-ribose) polymerase inhibitor; Ref, reference; SD, standard deviation.

**Table S6. Association between Ra-223 combination/layered therapy and real-world overall survival: unadjusted and adjusted Cox-regression models**

| Characteristic | Unadjusted model | | Adjusted model | |
| --- | --- | --- | --- | --- |
|  | HR (95% CI) | *P* value | HR (95% CI) | *P* value |
| Ra-223 combination/layered therapy (Ref = monotherapy) | 0.76 (0.64–0.90) | <0.05 | 0.78 (0.65–0.93) | 0.005 |
| Age groups (Ref = 18–64) |  |  |  |  |
| 65–74 |  |  | 1.54 (1.25–1.91) | <0.0001 |
| 75–79 |  |  | 1.35 (1.02–1.79) | 0.0335 |
| 80+ |  |  | 2.05 (1.57–2.69) | <0.0001 |
| Race (Ref = White) |  |  |  |  |
| African American |  |  | 0.73 (0.58–0.93) | 0.0121 |
| Other |  |  | 0.56 (0.43–0.74) | <0.0001 |
| Unknown |  |  | 1.29 (1.02­–1.62) | 0.0319 |
| Region (Ref = West) |  |  |  |  |
| Midwest |  |  | 1.10 (0.85–1.42) | 0.4792 |
| Northeast |  |  | 0.80 (0.62–1.03) | 0.0811 |
| South |  |  | 1.07 (0.83–1.37) | 0.6073 |
| CCI categories^a^ (Ref = ≥5) |  |  |  |  |
| 0 |  |  | 1.28 (0.96–1.71) | 0.09 |
| 1–2 |  |  | 1.19 (0.93–1.53) | 0.1593 |
| 3–4 |  |  | 0.96 (0.74–1.26) | 0.788 |
| Visceral metastases (Ref = No) |  |  | 0.91 (0.71–1.17) | 0.4608 |
| Ra-223 index LOT (Ref = 4+) |  |  |  |  |
| 1 |  |  | 0.42 (0.32–0.55) | <0.0001 |
| 2 |  |  | 0.58 (0.47–0.72) | <0.0001 |
| 3 |  |  | 0.75 (0.61–0.94) | 0.012 |
| Pain medication (Ref = No) |  |  | 1.36 (1.07–1.73) | 0.0127 |
| Baseline hospitalization  (Ref = No) |  |  | 1.32 (1.07–1.65) | 0.0114 |
| Baseline ED visit (Ref = No) |  |  | 1.15 (0.97–1.38) | 0.1143 |
| Baseline median outpatient visits >27 (Ref = ≤27 visits) |  |  | 1.19 (0.99–1.42) | 0.0621 |

^a^Excludes index conditions.

CCI, Charlson Comorbidity Index; CI, confidence interval; ED, emergency department, HR, hazard ratio; LOT, line of therapy.

**Table S7. Association between completion of ≥5 Ra-223 cycles and real-world overall survival: unadjusted and adjusted Cox-regression models**

| Characteristic | Unadjusted model | | Adjusted model | |
| --- | --- | --- | --- | --- |
|  | HR (95% CI) | *P* value | HR (95% CI) | *P* value |
| ≥5 Ra-223 cycles (Ref = 1–4 cycles) | 0.43 (0.37–0.51) | <0.0001 | 0.45 (0.38–0.53) | <0.0001 |
| Age groups (Ref = 18–64) |  |  |  |  |
| 65–74 |  |  | 1.49 (1.21–1.84) | 0.0002 |
| 75–79 |  |  | 1.40 (1.06–1.84) | 0.017 |
| 80–84 |  |  | 2.07 (1.58–2.70) | <0.0001 |
| Race (Ref = White) |  |  |  |  |
| African American |  |  | 0.74 (0.58–0.94) | 0.0142 |
| Other |  |  | 0.57 (0.43–0.75) | <0.0001 |
| Unknown |  |  | 1.27 (1.01–1.60) | 0.0383 |
| Region (Ref = West) |  |  |  |  |
| Midwest |  |  | 1.21 (0.94–1.57) | 0.1342 |
| Northeast |  |  | 0.86 (0.67–1.10) | 0.2333 |
| South |  |  | 1.10 (0.86–1.41) | 0.4611 |
| CCI categories^a^ (Ref = ≥5) |  |  |  |  |
| 0 |  |  | 1.16 (0.87–1.55) | 0.3097 |
| 1–2 |  |  | 1.19 (0.93–1.52) | 0.168 |
| 3–4 |  |  | 0.92 (0.71–1.21) | 0.5559 |
| Visceral metastases (Ref = No) |  |  | 0.95 (0.74–1.22) | 0.7011 |
| Ra-223 index LOT (Ref = 4+) |  |  |  |  |
| 1 |  |  | 0.47 (0.36–0.62) | <0.0001 |
| 2 |  |  | 0.63 (0.51–0.78) | <0.0001 |
| 3 |  |  | 0.82 (0.66–1.03) | 0.0827 |
| Pain medication (Ref = No) |  |  | 1.33 (1.05–1.70) | 0.0202 |
| Baseline hospitalization  (Ref = No) |  |  | 1.38 (1.11–1.72) | 0.004 |
| Baseline ED visit (Ref = No) |  |  | 1.14 (0.95–1.36) | 0.1599 |
| Baseline median outpatient visits >27 (Ref = ≤27 visits) |  |  | 1.15 (0.96–1.37) | 0.1323 |

^a^Excludes index conditions.

CCI, Charlson Comorbidity Index; CI, confidence interval; ED, emergency department, HR, hazard ratio; LOT, line of therapy.

**Fig. S1. Study design.**


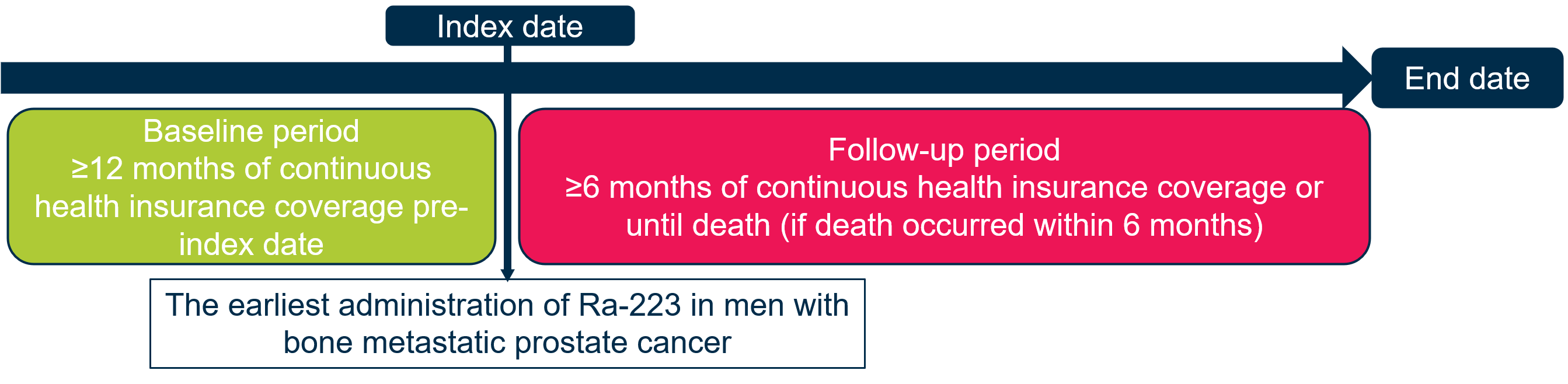


The identification period (between January 1, 2017 to June 30, 2022) was defined as the period identifying men with Ra-223 treatment initiation. The index date was the date on which individuals had the first evidence of a medical or prescription claim for Ra-223 during the identification period. The baseline period (≥12 months prior to the index date, excluding the index date itself) was used to identify the baseline demographic, clinical, medication use patterns, and other characteristics. The follow-up period (≥6 months from the index date until disenrollment, death, or end of study) was to assess treatment utilization patterns and follow-up medication use.
